# Clinical evaluation of the performance and safety of a new dentine substitute, Biodentine, in the restoration of posterior teeth — a prospective study

**DOI:** 10.1007/s00784-012-0701-9

**Published:** 2012-03-14

**Authors:** Gilles Koubi, Pierre Colon, Jean-Claude Franquin, Aline Hartmann, Gilles Richard, Marie-Odile Faure, Grégory Lambert

**Affiliations:** 1 Service d’Odontologie Gaston Berger, CHU de Marseille, 17-19 boulevard Mireille Lauze, 13010 Marseille, France; 2 AP-HP Service d’Odontologie Garancière-Rothschild, Université Paris Diderot, UMR 5615, Lyon 15, rue Santerre, 75012 Paris, France; 3 AP-HP Service d’Odontologie Garancière-Rothschild, Université Paris Diderot, 5 rue Santerre, 75012 Paris, France; 4 SEPTODONT, 58 rue du Pont de Créteil, 94107 Saint-Maur-des-Fosses, France

**Keywords:** Dental restoration, Dentine substitute, Dental cement, Biodentine, Tricalcium silicate-based cement

## Abstract

**Objectives:**

A multicentric randomized, 3-year prospective study was conducted to determine for how long Biodentine, a new biocompatible dentine substitute, can remain as a posterior restoration.

**Materials and methods:**

First, Biodentine was compared to the composite Z100®, to evaluate whether and for how long it could be used as a posterior restoration according to selected United States Public Health Service (USPHS)’ criteria (mean ± SD). Second, when abrasion occurred, Biodentine was evaluated as a dentine substitute combined with Z100®.

**Results:**

A total of 397 cases were included. This interim analysis was conducted on 212 cases that were seen for the 1-year recall. On the day of restoration placement, both materials obtained good scores for material handling, anatomic form (0.12 ± 0.33), marginal adaptation (0.01 ± 0.10) and interproximal contact (0.11 ± 0.39). During the follow-up, both materials scored well in surface roughness (≤1) without secondary decay and post-operative pain. Biodentine kept acceptable surface properties regarding anatomic form score (≤1), marginal adaptation score (≤2) and interproximal contact score (≤1) for up to 6 months after placement. Resistance to marginal discoloration was superior with Biodentine compared to Z100®. When Biodentine was retained as a dentine substitute after pulp vitality control, it was covered systematically with the composite Z100®. This procedure yielded restorations that were clinically sound and symptom free.

**Conclusions:**

Biodentine is able to restore posterior teeth for up to 6 months. When subsequently covered with Z100®, it is a convenient, efficient and well tolerated dentine substitute.

**Clinical relevance:**

Biodentine as a dentine substitute can be used under a composite for posterior restorations.

## Introduction

Amalgam, composites and glass-ionomer cements are commonly used to restore posterior teeth. The use of amalgam is decreasing due to poor aesthetic properties and concerns regarding mercury toxicity [1]. Composites fillings are becoming the reference for direct restorations since the current controversy on the abrasive effect of glass ionomers cements [2].

In posterior deep cavities, pulp health sometimes needs to be re-evaluated several months after a repair process. In such clinical situations, the biocompatible dentine substitute can be used first as a posterior restoration to obtain pulp healing. After validation of pulp health, it can be partially removed to place a permanent composite material in order to avoid bacteria exposure.

A new dental material, the tricalcium silicate based Biodentine could be both a temporary enamel restoration and a definitive dentine substitute. Its good sealing properties, high compression strengths and short setting time [3–5] are suggestive of its potential as a restorative material.

The aim of the study was to determine for how long Biodentine can remain as a restorative material submitted to occlusal chewing forces and how it can be managed in combination with a composite restoration. The resin-based composite Z100®, which has been in clinical use for the restoration of posterior teeth for more than a decade [6], was used as a comparator.

## Material and methods

### Study design and patients

This was a multicentric, randomized, prospective study which started in 2005, with a 3-year follow-up that was still ongoing at the time of this interim analysis. A total of 400 patients were estimated necessary to obtain at least 100 exploitable observations in each treatment group at M6 (6 months). Patients were recruited in the department of operative dentistry at two French university hospitals (Service d’Odontologie Gaston Berger, CHU de Marseille, Marseille and Service d'Odontologie Garancière-Rothschild, Paris). Ten investigators from two centers received training in Good Clinical Practice and on tooth models with Biodentine before the first inclusion. As the manipulation of Biodentine was not different from commonly used products, a special training of the investigators was not considered necessary. The protocol, its amendments and the informed consent form were approved by an Independent Ethics Committee (IEC Sud Méditerranée, France). The study was declared to the French Health competent authority (AFSSAPS) and registered under the reference number 2004/12/013. The study was conducted following the principles of the Declaration of Helsinki and the international consensus guideline (ICH) for Good Clinical Practice. Patients gave their informed, written consent prior to study enrolment.

Patients eligible to participate were between 18 and 80 years of age and had one or two indications for definitive occlusal or occluso-proximal restorations on vital posterior teeth from the first premolar to the third molar, with class I and II cavities, as evaluated with radiographs. A pulp vitality test was performed to exclude irreversible damage.

Non-inclusion criteria were non-vital teeth, contraindication to treatment with Biodentine or Z100® (3 M Z100® Restorative Dental Composite), allergy or hypersensitivity to an element of Biodentine or Z100®, serious periodontal problems, no desire for definitive restoration, and previous treatment with radiotherapy.

### Study procedures and study outcome

Patients were randomly assigned to receive either Biodentine or the comparator product Z100® using Microsoft® Office Excel® software. Restoration was performed on D0 (day 0). Cavity preparation was carried out with a diamond bur (shape 830) according to the requirements for a direct composite restoration.

For the Z100® group, an adhesive system (All Bond 2, Bisco, IL, USA) was applied and then light-cured for 10 s, using a daily controlled light curing unit (Satelec, F, 800 mW/cm^2^). After repeated air flow, Z100® was applied using a horizontal incremental placement technique (maximum: 2 mm thickness layer). Increments were light-cured for 20 s according to the manufacturer’s instructions.

Biodentine is a high purity Ca_3_SiO_5_-based dental material (Laboratoires Septodont, Saint-Maur, France) composed of a solid part containing tricalcium silicate (3CaO·SiO_2_), calcium carbonate (CaCO_3_) and zirconium oxide (ZrO_2_) and a liquid part containing calcium chloride (CaCl_2_·2H_2_O), and a water reducing agent [4]. Both parts were provided in single-dose units. Five drops of liquid were added to the powder single unit. After mixing 30 s at 4,000–4,200 rpm, Biodentine was applied as such without requiring any surface treatment.

A control radiograph was taken after the procedure. Follow-up visits were scheduled for D15, M6, M12, M24, and M36 and included a clinical evaluation and a radiographic examination.

The study outcomes were material performance and material safety and were assessed at each visit. Material performance was assessed by evaluating characteristics of the product application, including consistency, working time (i.e., whether the duration for preparation and application the material was adequate), adhesion to instruments, and ease of handling. Furthermore the characteristics of the restoration, including anatomic form of the restoration, marginal adaptation, quality of proximal contact, marginal discoloration, surface roughness, secondary decay, and post-operative pain were analysed.

Since the main objective was to evaluate for how long Biodentine could be used as a long term temporary posterior restoration, criteria were selected from the USPHS. However, in this indication some criteria were not relevant.

The following scoring scales were used for these evaluations:

- Consistency, working time, adhesion to instruments, ease of handling (0 to 3): 0= very satisfying; 1= satisfying; 2= unsatisfying, 3= very unsatisfying.
- Anatomic form (0 to 3): 0= restoration is continuous with existing form; 1= restoration is discontinuous, without exposure of the dentine or base; 2= part of restoration missing, enough to expose the dentine; 3= partial or total restoration loss, fracture, traumatic occlusion, pain in tooth or surrounding tissue. A score above 1 was considered clinically unacceptable.
- Marginal adaption (0 to 4): 0= complete adaptation of the restoration to the tooth, no visible marginal defects; 1= significant defects, but no dentine exposure; 2= significant marginal defects with dentine exposure; 3= fractured and mobile restoration, insufficient material; 4= restoration mobile, fractured or lost. A score above 2 was considered clinically unacceptable.
- Quality of proximal contact (0 to 2): 0= presence of contact, but insufficient space to pass dental floss between the tooth and the restoration; 1= presence of contact; sufficient space to pass dental floss between proximal tooth and restoration; 2= no contact between the tooth and the restoration. A score of 2 was considered clinically unacceptable. In case of class I restoration, this evaluation was not performed.
- Marginal discoloration (0 to 3): 0= no discoloration; 1= slight discoloration; removed with polishing; 2= obvious discoloration; not removable with polishing; 3= considerable discoloration. A score above 2 was considered clinically unacceptable.
- Surface roughness (0 to 3): 0= smooth surface; 1= slightly rough; 2= rough; no new finish allowed; 3= deep pitted surfaces, irregular fissures. A score above 1 was considered clinically unacceptable.
- Secondary caries (0 to 1): 0= no evidence of marginal caries; 1= visible marginal caries. A score of 1 was considered clinically unacceptable.
- Post-operative pain (0 to 2): 0= no pain; 1= acceptable pain; 2= unacceptable pain. A score of 2 was considered clinically unacceptable.

Safety analysis involved the evaluation of adverse events at each visit.

### Z100***®*** covering treatment

The provision was made in the protocol that patients who required reconstitution of an aged restoration, could remain in the study if some of the initial restorative material was left in place, to cover the base of the cavity. The remaining cavity was to be filled with Z100® using the closed sandwich technique. The same adhesive system, as for the Z100®’s group, was applied both on dental tissues and Biodentine.

### Statistical analysis

The results reported here are derived from an interim analysis. Included were the data of all patients who participated in the study at least until the follow-up visit at M12. The evaluation criteria were analysed using the scales described above. If one of the criteria received an unacceptable score, the restoration was considered a failure and a new Z100® restoration was placed. The scores of the two restorative materials were compared using the following non-parametric tests: Friedman test complemented with Wilcoxon *t*-test to evaluate the influence of time on the material (correction of p-values for multiple comparison); Mann–Whitney *U*-test to evaluate the material at each time point as chi-square frequency method criteria were not fulfilled. All tests were two-sided with a significance level of 5%. Results are shown as mean ± SD (standard deviation).

## Results

### Restoration procedure

At the time of the interim analysis, patient recruitment was ended and 397 cases were included in the clinical study. On these 397 cases, 212 cases were restored and followed for at least 12 months. Results presented here concerned these 212 cases. Cases were distributed in 77 (36.3%) Class I cavities and 135 (63.7%) Class II cavities. The distribution between molars and premolars was 64 (30.2%) and 148 (69.8%), respectively. Ninety-six patients with a mean age of 36 (±14) years received a restoration with Biodentine and 116 patients with a mean age of 39 (±14) years received Z100®. The restorative procedure was similar in both groups, with the exception that more patients in the Biodentine group required matrix placement and needed retention form, but no cavity liners were applied in this group.

### Restoration performance

After placement of the restorations on D0, both products received scores for material handling between 0 and 1, i.e., very satisfactory and satisfactory (Table 1). The consistency score was statistically superior for Biodentine compared to Z100® (*p* = 0.005). The characteristics of the restoration in place, including anatomical form, marginal adaptation and interproximal contact were rated as very satisfying by the investigators (mean scores near 0).

**Table 1:** Material performance and clinical evaluation of the restoration on D0

	Biodentine (*N* = 96)^a^	Z100**®** (*N* = 116)^b^
Material handling (mean (±SD); min, max)		
Consistency	0.54 (±0.92); 0, 3	0.17 (±0.40); 0, 2
Working time	0.55 (±0.98); 0, 3	0.25 (±0.47); 0, 2
Non adhesion to instruments	0.37 (±0.65); 0, 2	0.43 (±0.56); 0, 2
Ease of handling	0.64 (±1.02); 0, 3	0.25 (±0.45); 0, 2

Clinical evaluation of the restoration (mean (±SD); min, max)		
Anatomic form	0.12 (±0.33); 0, 1	0.05 (±0.22); 0, 1
Marginal adaptation	0.01 (±0.10); 0, 1	0.02 (±0.13); 0, 1
Interproximal contact	0.11 (±0.39); 0, 2	0.01 (±0.12); 0, 1

^a^For 22 patients, data were missing for interproximal contact

^b^For 47 patients, data were missing for interproximal contact

**Fig. 1:**
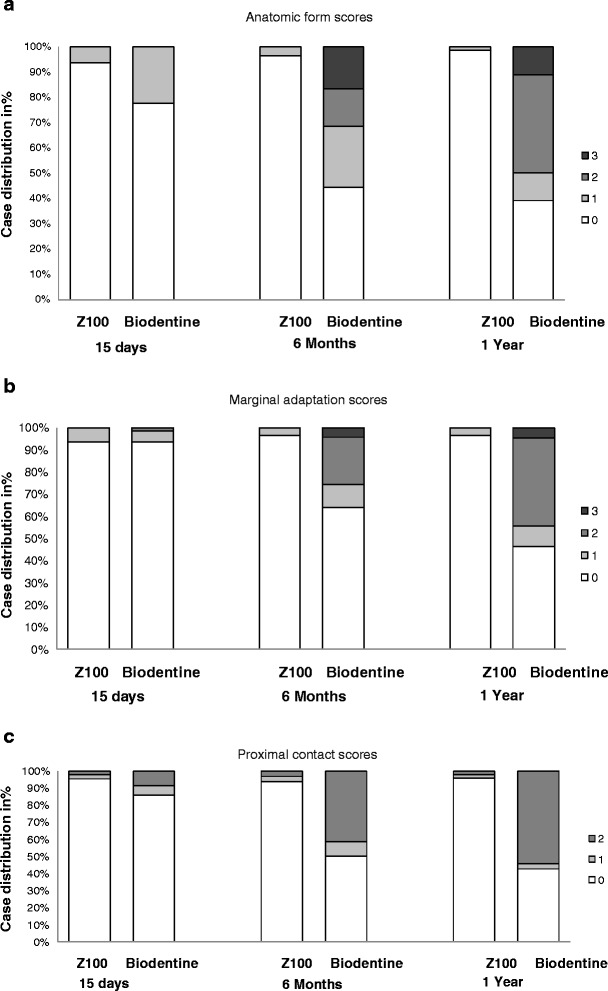

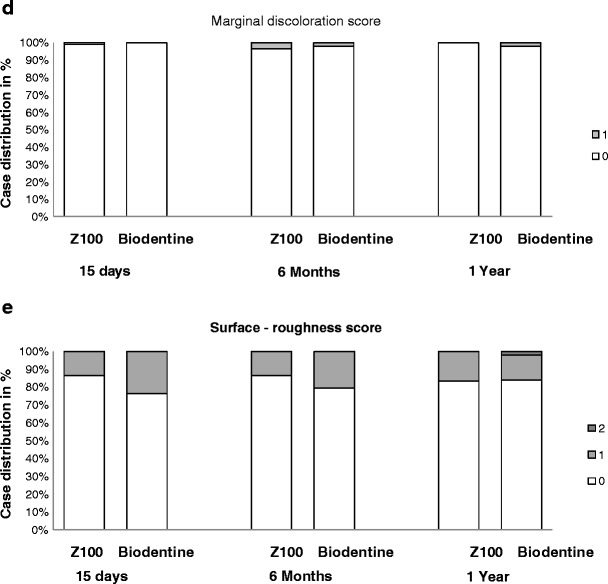
Restoration evaluation during the follow-up phase: anatomic form (**a**), marginal adaptation (**b**), quality of proximal contact (**c**), marginal discoloration (**d**), and surface roughness (**e**). Shown is the percentage of patients who were attributed the indicated scores at the follow-up visits D15, M6 and M12. The following scores were statistically significantly different between Biodentine and Z100® restorations: for “anatomic form” at each time point (*p* = 0.004 on D15, *p* < 0.001 at M6 and M12); for “marginal adaptation” and “point of proximal contact” at M6 and M12 (*p* < 0.001). The scores for “marginal discoloration” and “surface roughness” were not significantly different

During the follow-up period, product performance was evaluated at each visit. The anatomic form score remained very satisfactory for the majority of patients with Z100® restorations throughout the study. In Biodentine group, the product achieved acceptable scores up to 6 months after the restoration for the anatomic form, the marginal adaptation and the proximal contact. The difference at 6 months and at 1 year between the two groups was statistically significant (Fig. 1a–c).

Marginal discoloration and surface-roughness scores remained very satisfactory in both treatment groups throughout the study (Fig. 1d–e). There was one case of secondary caries reported at M6 in the Biodentine group due to the restoration loss 2 months prior to the visit.

### Z100***®*** covering treatment

**Fig. 2:**
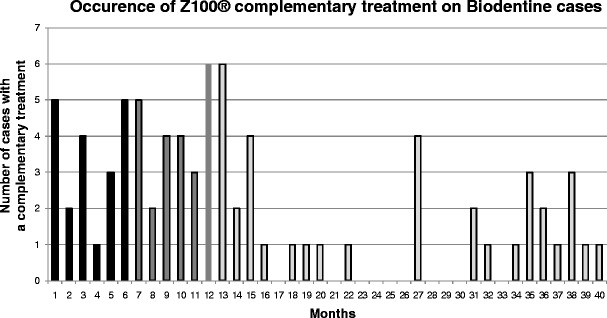
Occurrence of Z100® complementary treatment on Biodentine cases. Shown is the number of Biodentine cases recovered by Z100® related to the date (expressed in month) when occurred the complementary treatment of Z100®. Black bars represent 1–6 months, grey bars represent 7–12 months and the light grey bars represent after 12 months

At the time of the analysis, Z100® demonstrated better scores for anatomic form, marginal adaptation and proximal contact than Biodentine at the 6-month visit. This situation was confirmed at 1-year visit. During the follow-up of the first cases, it appeared that Biodentine was abraded and required an additional restoration with Z100®. At the interim evaluation, results showed that 80 cases underwent an additional restoration. Twenty-five percent of cases occurred before the 6-month visit, 30% between 6 months and 1 year and 46% after 1 year (Fig. 2). The reason for placing a composite over the Biodentine was mainly abrasion (on the occlusal surface or on the proximal contact). Details are listed in Table 2. It was decided to systematically add the composite to the Biodentine at the next visit for the other cases. Therefore, the second step of the study was to determine the efficacy of Biodentine covered with Z100®. It was found that the Biodentine restoration was well tolerated as all patients tested had a positive pulp vitality test. Biodentine was cut back using dental burs, but preserved as a dentine substitute in order to avoid bacterial infiltration through dentine tubules. The resistance of Biodentine to be cut back was found to be very satisfactory. Almost all investigators rated the complementary treatment procedure as very satisfactory. The evaluation of anatomic form, marginal adaptation, proximal contact, the resistance to marginal discoloration, surface roughness, the absence of secondary caries, and post-operative pain yielded very satisfactory scores throughout the study.

**Table 2:** Complementary treatment in patients with Biodentine restorations (*N* = 80)

Reasons for complementary treatment (%)	
Abrasion	88.8
Quality of proximal contact	65
Fracture	21.3
Initial indication	8.8
Esthetics	6.3
Biologic	1.2
Other	2.5

Initial Biodentine remaining (%)	93.8^a^
Good resistance to burring (%)	93.8^a^

^a^The remaining 5.2% (3 patients) had lost their initial Biodentine restoration

### Material safety

At the time of the interim analysis, a total of eight adverse events were reported on 212 cases, four cases in each group. In the Z100® group, there was one case of a mouth ulcer, which was probably not related to the product, three cases of pain (one was persistent and certainly imputable to the adhesive/composite system which was removed, the other two lasted 3 days maximum and were probably related to the adhesive/composite system). In the Biodentine group, there was one case of cold sensitivity due to the loss of a part of the material on the occlusal surface and three cases of pain of which one was probably not imputable to the product (failure during the diagnosis).

## Discussion

This was the first clinical trial evaluating the performance and safety of Biodentine, a new dentine substitute composed mainly of tricalcium silicate [3]. The biocompatibility of this material was recently proven in in vitro and in vivo studies [3, 4]. Importantly, the material did not affect human pulp fibroblast specific functions such as mineralization, as well as expression of collagen I, dentine sialoprotein and Nestin [3, 7–9]. Biodentine may enhance the repair and pulp healing in case of partial impairment of the odontoblastic layer. Given the mechanical properties of Biodentine, we expected it to be a posterior restorative material in clinical situations where the evaluation of pulp healing is required before a definitive restoration.

In the present trial, Biodentine received good rates for material handling and performance after restoration placement. Two evaluation criteria with excellent ratings for both materials were absence of post-operative pain and secondary decay. Post-operative pain is frequently observed in class I and II restorations in posterior teeth with resin-based materials [10]. In our study almost all patients analysed were free of post-operative pain. Only one case of secondary caries was reported in the Biodentine group after a 1-year follow-up. This case was due to the loss of the material. No other adverse events were observed after Biodentine application. Another common problem observed in posterior composite restorations is marginal discoloration [11]. We found that Biodentine had significantly better scores for this characteristic compared to Z100®. However, it has to be kept in mind that this is an interim study and that only a subset of patients had completed the 3-year follow-up.

At this interim analysis, Z100® had better scores for anatomic form, marginal adaptation and proximal contact than Biodentine at the 6-month recall. This situation was confirmed at the 1-year recall. Moreover, during the follow-up of the first cases, it appeared that some abrasion process occurred on Biodentine restorations for 25% before 6 months and 30% between 6 months and 1 year. Although the deficiencies of marginal adaptation required a new restoration, no marginal discoloration occurred. Therefore, it was decided to systematically add the composite on top of Biodentine for the next visit in the other cases. Then, it was the second step of the clinical study: the evaluation of Biodentine as a dentine substitute combined with direct composite restoration.

When Biodentine was used as a dentine substitute combined with direct composite restoration, we found it to be easily cut back with a dental bur at partial removal stage. A thick layer was left in place as a dentine substitute in order to preserve biological and sealing effects [12, 13]. The addition of Z100® was carried out using the sandwich technique. This may constitute a therapeutic advantage for patients, especially for large cavities where the risk of secondary caries is increased [14]. This approach may help to preserve tooth structure and improve the longevity of the restoration. Importantly, this treatment resulted in very satisfactory restoration performance.

To conclude, regarding handling properties and behavior in stress bearing conditions of posterior teeth, Biodentine can be successfully used as a posterior restoration material for up to 6 months. At this time, abrasion is the main degradation process without any marginal discoloration. Thus, the clinical relevance of this study is the ability to use Biodentine as a dentine substitute under a composite for posterior restoration.
